# A cluster randomised controlled trial of a ward-based intervention to improve access to psychologically-informed care and psychological therapy for mental health in-patients

**DOI:** 10.1186/s12888-022-03696-7

**Published:** 2022-02-03

**Authors:** Katherine Berry, Jessica Raphael, Helen Wilson, Sandra Bucci, Richard J Drake, Dawn Edge, Richard Emsley, Gill Gilworth, Karina Lovell, Bolanle Odebiyi, Owen Price, Matt Sutton, Rachel Winter, Gillian Haddock

**Affiliations:** 1 Division of Psychology and Mental Health, School of Health Sciences, Faculty of Biology, Medicine and Health, Manchester Academic Health Sciences, The University of Manchester, Manchester, UK; 2 Greater Manchester Mental Health NHS Foundation Trust, Manchester, UK; 3 Institute of Psychiatry, Psychology and Neuroscience, King’s College London, London, UK; 4 Division of Nursing, Midwifery and Social Work, School of Health Sciences, Faculty of Biology, Medicine and Health, The University of Manchester, Manchester, UK; 5 Division of Population Health, Health Services Research & Primary Care, The University of Manchester, Manchester, UK

**Keywords:** Cluster trial, Psychological therapy, Acute mental health, Inpatient, Process evaluation, Health economic analysis

## Abstract

**Background:**

There is good evidence that psychological interventions improve patient well-being and independent living, but patients on acute mental health wards often do not have access to evidence-based psychological therapies which are strongly advised by NICE guidance for severe mental health problems. The overall aim of this programme of work is to increase patient access to psychological therapies on acute mental health inpatient wards. Stage one of the programme (which is complete) aimed to identify barriers and facilitators to delivering therapy in these settings through a large qualitative study. The key output of stage one was an intervention protocol that is designed to be delivered on acute wards to increase patient access to psychologically-informed care and therapy. Stage two of the programme aims to test the effects of the intervention on patient wellbeing and serious incidents on the ward (primary outcomes), patient social functioning and symptoms, staff burnout, ward atmosphere from staff and patient perspectives and cost effectiveness of the intervention (secondary outcomes).

**Methods:**

The study is a single blind, pragmatic, cluster randomised controlled trial and will recruit thirty-four wards across England that will be randomised to receive the new intervention plus treatment as usual, or treatment as usual only. Primary and secondary outcomes will be assessed at baseline and 6-month and 9-month follow-ups, with serious incidents on the ward collected at an additional 3-month follow-up.

**Discussion:**

The key output will be a potentially effective and cost-effective ward-based psychological intervention that increases patient access to psychological therapy in inpatient settings, is feasible to deliver in inpatient settings and is acceptable to patients.

**Trial Registration number:**

ClinicalTrials.gov Identifier: NCT03950388. Registered 15^th^ May 2019. https://clinicaltrials.gov/ct2/show/NCT03950388

## Background

Severe mental health problems, such as schizophrenia and bipolar disorder, are characterised by profound disruptions in thinking, perception and everyday functioning [1]. More than 1.5 million people in England have a severe mental health problem. Symptoms and difficulties in functioning often start in young adulthood and can have a hugely distressing impact on the individual, family and friends. The care of people with severe mental health problems costs around £12 billion a year. This figure accounts for more than half of the mental health and social care budget and much of it is spent on acute inpatient admissions [2].

Between April 2017 and March 2018, 103,952 people who were in contact with mental health services spent time on mental health wards [3]. On average, it costs the NHS over £12,000 per acute inpatient admission [2]. Despite extremely high costs associated with inpatient care, inquiries and surveys of patient satisfaction highlight poor quality of care in these settings [1, 4, 5] and there are high levels of staff burn out and absenteeism [6].

Surprisingly, inpatients do not typically have access to evidenced-based psychological therapies, which are both wanted by patients and strongly advocated by NICE guidance for severe mental health problems, including Schizophrenia, Bipolar Disorder, Major Depression and Personality Disorder [7–10]. On the back of significant lobbying by patient and carer groups, the Mental Health Task Force [11] now advocates a ‘referral to treatment access’ standard for psychological therapy in acute inpatient care, meaning that NHS Mental Health Trusts will need to deliver timely, evidence-based psychological treatments in these settings.

Previous reviews demonstrate the benefits of psychological therapies for severe mental health problems, in terms of improvements in symptoms and reduced costs to the NHS [2, 12, 13]. However, recent systematic searches have found that very few trials of psychological therapies target inpatient wards [14]. Globally, at the time of writing a total of 68 studies have been carried out specifically in acute inpatient settings, but the majority of the studies are medium to low quality with low sample sizes, non-randomised designs, no blind assessment of outcomes, or not reporting -intention to treat analyses. Even when researchers use randomised designs, they often demonstrate poor randomisation methods and allocation concealment, with only two randomised control trials utilising an independent randomisation service and a large number not specifying the randomisation method at all. Furthermore, many studies target one particular type of problem only or specific diagnostic groups, such as early psychosis, depression or self-harm, thus excluding a significant proportion of patients on the ward. Although there is good evidence for psychological therapies from high quality trials with outpatients, we propose that existing evidenced-based psychological therapies need to be adapted for inpatient settings. The process of delivering therapy in inpatient settings is likely to present unique challenges which require empirical investigation. For example, patients are likely to be experiencing higher levels of distress and the relatively short length of patient stay may impact on the potential length of therapy. Additionally, there is an increased need to consider attitudes and relationships with other mental health workers. Relatedly, with the exception of our own study, which found a positive effect for Cognitive Behaviour Therapy compared to a control therapy on violence and risk management [15], most therapy trials have used patient symptoms as the primary outcome. We argue that serious incidents or so-called risky behaviours are more important outcomes to target and assess in inpatient settings because they: a) are more prevalent in inpatient settings; b) they prevent patients being discharged and therefore prolong length of stay; c) are highly distressing for all those involved, including patients themselves, carers, staff and other patients; and d) are extremely costly due to their impact on length of stay and the resource needed to manage them [16]. Despite the importance of serious incidents for inpatients, as research has focused on outpatient therapy, there is more limited evidence for the effects of psychological therapy on reducing serious incidents.

Given these gaps in knowledge, the first stage of this programme of research aimed to investigate barriers and facilitators to delivering therapy in inpatient settings with the end goal of developing an intervention protocol to maximise access to psychological therapy and psychologically-informed care on inpatient settings. This research took the form of: a) a review of 51 studies implementing psychosocial interventions on acute mental health wards [17]; b) a large qualitative study of 26 staff, 22 patient and 12 carer perspectives on inpatient therapy using semi-structured interviews; and c) two pilot studies where we implemented draft intervention protocols and used interviews to elicit stakeholder views on what aspects of the intervention and its delivery worked or did not work in practice [18]. The findings have resulted in the development of a model of how to deliver therapy on patient wards called TULIPS (Talk, Understand and Listen for In-Patient Settings). Key elements of the TULIPS model were agreed and prioritised during an expert consensus conference where nationally-drawn stakeholders, including national leaders in the development of psychological therapy for severe mental health problems, senior clinicians and managers working in inpatient setting and patient/carer representatives were asked to vote on questions posed by the research team [19]. Here we describe a protocol to evaluate the TULIPS intervention compared to treatment as usual, using a cluster randomised control trial. Anticipated benefits of the intervention are reductions in serious incidents on the ward, improved patient well-being and functioning and reduced staff burnout.

## Objectives

The primary objectives are: i) to assess the effectiveness of a psychological service model designed specifically for acute mental health wards; and ii) determine whether it reduces the occurrence of ward-level serious incidents and improves patient wellbeing. Secondary objectives are to: assess whether the intervention improves patient and staff perceptions of ward atmosphere, staff burnout, patient mental health symptoms and functioning; and to explore patient resource use to assess the cost-effectiveness of the psychological service model.

## Methods

### Trial design

The study is a single blind, pragmatic, cluster randomised controlled trial. The TULIPS model will be delivered at the ward level (cluster) and compared to treatment as usual within a superiority framework, using a one-to-one allocation ratio, with minimisation based on Trust and ward gender (male/female/mixed). The trial will include a 9-month internal pilot which will have clearly defined stop/go criteria. The comparator was selected on the basis of pragmatics and difficulty in identifying a suitable control comparator.

### Blinding and Randomisation

Once a site is recruited all eligible and consenting wards will be randomised. To maintain allocation concealment, the allocation will be conducted by an independent statistician at the King’s Clinical Trials Unit. Wards will be identified, and their Trust and ward gender (male/female/mixed gender) recorded. These will be entered into the minimisation program. If it does not make any difference to the balance on these variables which group is chosen, the ward will be allocated randomly. Otherwise, it will be allocated to the group which would make the two groups better balanced on these variables.

Members of the Trial Steering Committee (TSC), study statisticians, health economists and outcome assessors will be blinded to treatment allocation while the trial is ongoing. If the outcome assessors know (or suspect) they have been unblinded, this will be recorded on an unblinding form. For practical reasons linked to the setup and delivery of the intervention, some members of the research team and will not be blinded, including the Project Manager and Chief Investigator. Due to the nature of the intervention, participants and intervention delivers will not be blinded following the commencement of the intervention. Ward allocation will however, be concealed from potential participants until the completion of baseline measures to reduce the risk of selection bias.

### Participants

**Fig. 1:**
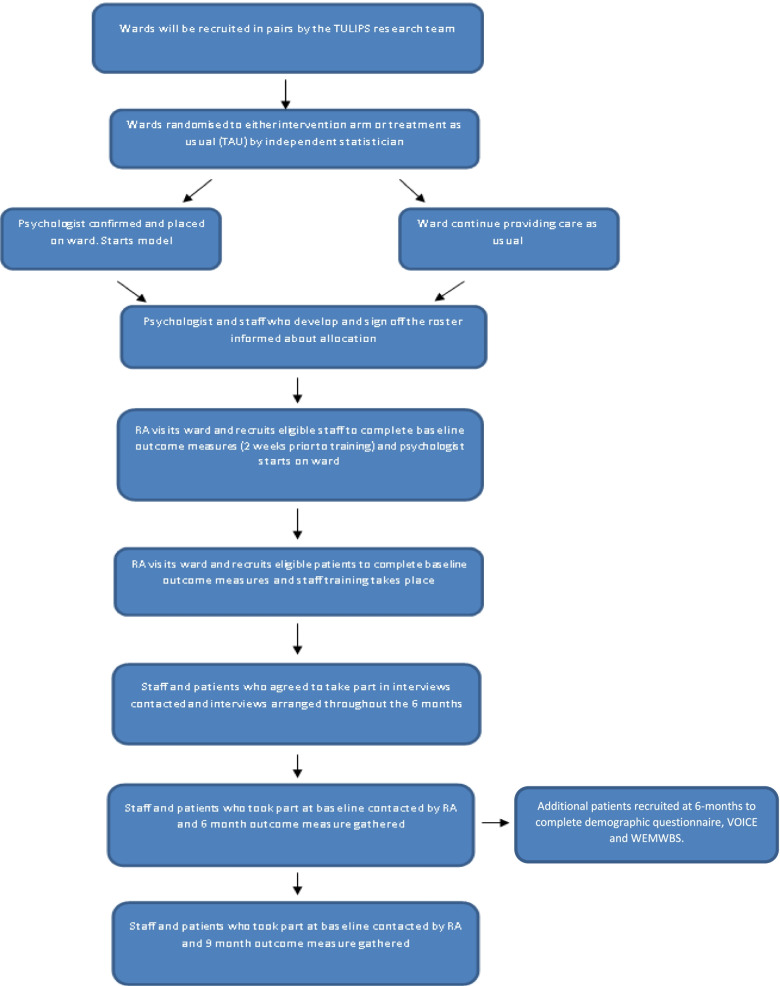
Participant flow through study

**Fig. 2:**
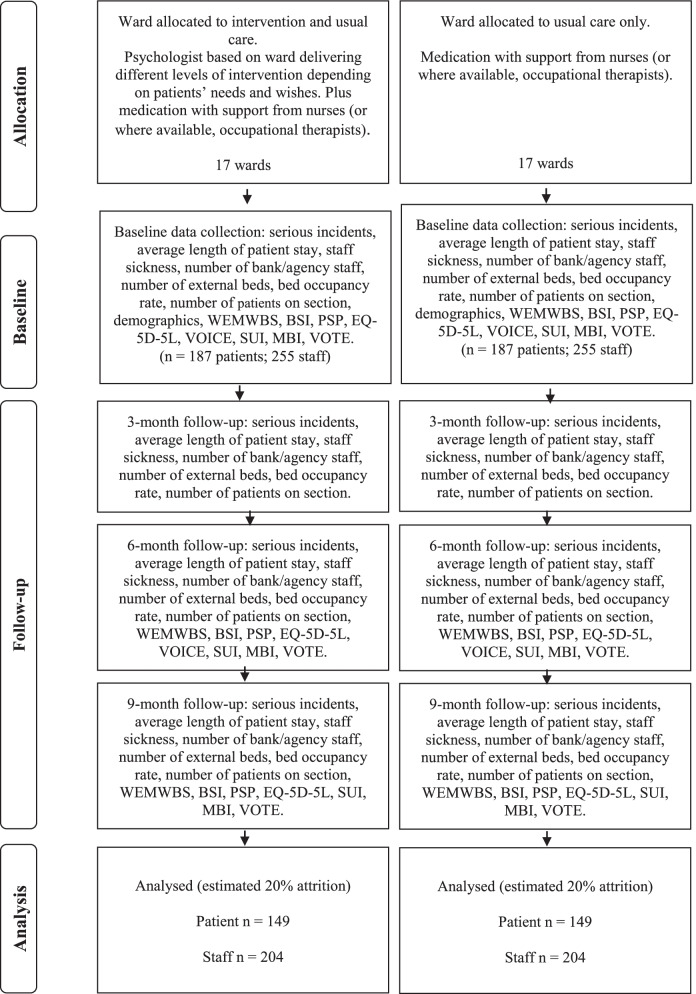
Consort diagram

Participants will include patients and staff who are based on an acute mental health ward recruited to the study. See Fig. 1 for participant flow through the study. See Fig. 2 for consort.

## Eligibility criteria

### Ward eligibility

Inclusion criteria for wards will be acute mental health wards. Exclusion criteria will be: wards that have a specialist function, such as organic impairment, intensive care or rehabilitation; and wards that already have more than two sessions of dedicated psychological therapy input per week. This latter criterion is to ensure treatment as usual does not include significant elements of the intervention we are aiming to test.

### Participant eligibility

We will apply the following criteria to identify suitable patient participants for each aspect of the study**:**

- informed consent
- sufficient levels of concentration and English language to complete the required battery of self-report measures with breaks if needed.

These criteria will be determined by the researcher when meeting the participant and in collaboration with the clinical team.

We will exclude:

- patients whose discharge is planned for before the start of the intervention period
- staff who plan to leave their jobs during the intervention time period
- agency workers, other non-permanent members of staff unless they are a regular bank worker or people who work less than 75% of their working week on the ward.

### Site selection and participant recruitment

Thirty-four generic acute mental health wards will be recruited across England and Wales. The sites will be selected across geographically diverse locations and include Greater Manchester Mental Health NHS Foundation Trust where the study is based.

### Recruitment

The baseline data will be gathered after the ward has been randomised. This is not the optimal design as it runs the risk of selection bias and in particular the control wards not being motivated to engage in the intervention. However, it was deemed to be the most practical solution due to difficulties in organising training for all ward staff at the start of the intervention (at least 4 weeks are needed to schedule rosters, backfill ward staff and book suitable rooms) and the importance of recruiting patients just prior to the start of the intervention (so they are exposed to at least some elements of it before being discharged from the ward). In order to circumvent the risks associated with early randomisation, the outcome of the randomisation will only be disclosed to a few key people (see below).

During the ward recruitment period, all staff on the ward and all patients on the ward meeting inclusion criteria will be approached about the study. Researchers will base themselves on the wards during the recruitment periods in order to promote the study and maximise opportunities for recruitment. Staff and patients will be provided with a written copy of the participant information sheet describing the details of the study. Where possible, potential participants will be given at least 24 hours to decide whether or not they would like to participate. However, if a participant wants to take part before this 24-hour window, we will give them the opportunity to do so. In all instances, participants will be provided with the opportunity to read the participant information sheet, be provided with the opportunity to ask questions about the research, and have their questions answered satisfactorily. They will be asked to consent to completing self-reported outcome measures and/or consent to take part in a qualitative interview. The information sheet and consent form will state that their participation is entirely voluntary and that they are free to withdraw from the study at any point. It is likely that most of the patients recruited at baseline will be discharged before the follow-up period. Therefore, follow-up measures will be completed in community settings. Researchers will take a proactive approach to follow-up assessment which will involve multiple attempts to contact people and being flexible with appointment times and locations, including the possibility of conducting follow-ups via email/ Microsoft Teams (staff) and/or phone (staff and patients).

Separate consent will be sought from patients on the ward at 6-month follow-up to complete the Views on Inpatient Care (VOICE; [20]). This is because it is likely different patients who will need to be recruited from baseline given the probability that the same patients will no longer be on the ward. Although participants will not be blind to the treatment condition of the ward, there will be a separate participant information sheet for the VOICE, which will not explicitly refer to the fact that the study is testing the effects of providing psychological therapies on inpatient wards. Potential participants will be informed that the study is assessing patient satisfaction with wards with different compositions of multidisciplinary team members**.**

### Intervention

All patients on the ward during the intervention period will be involved with a stepped model of care intervention at one of three levels. The level the patient receives will be decided by the multidisciplinary ward team, considering the patient’s and carers’ wishes and needs at the time. At Step one, all patients will have a psychological formulation developed by a Band 8a or 8b Health and Care Professions Council registered psychologist in conjunction with the patient or members of the ward team (which will include the person’s named nurse). A formulation provides a framework for bringing together biological, societal, cultural and psychological factors that might be responsible for the development and maintenance of problems and thus facilitate planning and implementing the most appropriate intervention [21]. The formulation will focus on the development or maintenance of difficulties depending on needs of those involved and the information available. No specific therapeutic model is stipulated on the basis that different psychologists may be trained in different approaches and the process of formulating is deemed to be more important than the specific methodology. Discussions arising from the formulation at Step one will determine whether or not the patient would benefit from being offered a Step two or three intervention. To help support this work, all ward staff will undertake a half-day training session at the beginning of the intervention period (ideally within the first two weeks). This initial training session will describe psychosocial models of mental health and the role and process of formulation.

At Step two all qualified nurses will be trained and supervised by a psychologist to deliver guided self-help material of psychological interventions targeting key problem areas for patients (e.g. anxiety management, behavioural activation for low mood, coping with symptoms of psychosis, minimising self-harm behaviours and suicidality). The staff will be provided with guided self-help booklets to use with patients and be requested not to use their own material. Interventions can be delivered in group or one-to-one formats. The level of group versus one-to-one sessions is to be determined in collaboration with senior ward staff. All qualified nursing staff will be trained in the guided-self-help material at the beginning of the intervention (ideally within the first two weeks) and will receive fortnightly supervision slots with the psychologists to support any work they are undertaking.

At Step three, patients who are felt to have needs that cannot be met at Step 2 and who want to engage in psychological therapy will be offered up to 16, one-to-one therapy sessions with the psychologist. These sessions will most likely focus on understanding the reasons for the current or repeated relapses and developing coping tools to address key factors triggering relapse. The length and frequency of each session will be determined on a case-by-case basis taking into account the patient’s needs and wishes, as well as the views of the wider multi-disciplinary team. No model of therapy is specified on the basis that the psychologist may be trained in different approaches and the TULIPS intervention is primarily testing the delivery a psychologically-informed environment as opposed to testing a specific form of therapy. However, the sessions should rate highly on non-specific therapeutic factors and working alliance.

In addition to delivering the stepped model of care, the psychologist will also deliver fortnightly group-based, staff reflective practice sessions of one-hour duration and fortnightly team formulation sessions of one-hour duration. Note that these team formulation meetings are separate and in addition to level one formulations and are about a group of staff coming together to reflect on their current and future work with a patient. Decisions about which patients to discuss during these meetings should be based on staff request. However, the psychologist should ensure that meetings do not focus solely on high risk patients as those with lower risk who are less problematic for staff may be excluded. The psychologist will also attend ward MDT meetings. The number of MDT meetings attended, and frequency will be determined in collaboration with senior nursing and medical staff.

The intervention period will run over a period of 6-months and wards randomly assigned to receive the intervention will employ a Psychologist NHS Band 8a or 8b on the ward for 0.5FTE for 7 months to support intervention delivery.

No restrictions will be placed on other care and interventions that are delivered during the trial, but we will attempt to monitor other interventions delivered to patients including other psychological intervention as part of the patient resource use inventory (see outcome measures section below). We will also record any dedicated psychological resource for both intervention and control wards in the study in terms of whole-time equivalent resource and banding.

### Outcome measures

Co-primary outcome measures have been selected. The first measure is total number of serious incidents (risky behaviours) per ward. We will be collecting serious incidents at baseline, 3-month follow-up, 6-month follow-up and 9-month follow-up. The data will be requested for the prior 3 months and will include incidents that fall into the categories of self-harm and violence (to staff, patients, and others).

All Trusts involved in the study are already required to collect ward level data on serious incidents as part of standard practice. Reporting criteria and procedures for serious incidents will be reinforced by senior managers in both intervention and control wards recruited to the study to sensitise all participating wards to reporting risk. As an additional protection against bias, blind assessors will collate data of staff-reported serious incidents and complete a 25% check across the 34 wards to ensure that they meet well operationalised definitions utilised in our previous research [15, 22].

The second primary outcome measure will be patient well-being as assessed by the Warwick-Edinburgh Mental Well-being Scale (WEMWBS; http://www2.warwick.ac.uk/fac/med/research/platform/wemwbs), which is a patient-reported health measure that will be collated at the individual patient level by blind assessors. The WEMWBS is used widely by IAPT for SMI and previous studies show that it is rated favourably by patients [23].

Despite the argument for the importance of serious incidents, we recognise the equally important value of targeting and assessing patient-reported outcome measures and will therefore measure patient mental well-being.

Other important outcomes to target and assess are distressing mental health symptoms and level of patient social functioning. These will be measured through the Brief Symptom Inventory (BSI; [24]) and Personal and Social Performance Scale (PSP; [25]). Improving these outcomes is not only important for patients and their families, but is likely to reduce length of admission, which in turn has the potential for significant cost savings. A further important outcome is patients’ perceptions of ward atmosphere [7, 11, 26] and previous research has suggested that working with ward staff to help them to deliver more psychologically-informed care can improve patient perceptions of ward atmosphere [27]. Improving patient access to psychological therapies is also likely to have a positive effect on frontline mental health staff who often experience high levels of burnout when working with risky behaviours. Patient and staff perceptions of the ward atmosphere will be assessed using the Views on Inpatient Care (VOICE; [20]) and Views on Therapeutic Environments (VOTE; [28]), respectively.

Staff burnout will be measured using the Maslach Burnout Inventory (MBI; [29]). Additional ward level data, patient resource use, and health-related quality of life measures will support the health economic evaluation.

See Table 1 for further information on who completes which measures in the study and at what time point.

**Table 1:** Outcome measures and time-points for collection

**Measures**	**Baseline**	**6 Month** **Follow Up**	**9 Month** **Follow Up**
**Patient Measures**			
Informed consent	x	x (VOICE participants)	
Demographics	x	x (VOICE participants)	
Brief Symptom Inventory (BSI; Derogatis, 1993)	x	x	x
Personal and Social Performance Scale (PSP; Morosini et al, 2000)	x	x	x
Quality of Life (EQ-5D-5L)	x	x	x
Views on In-Patient Care (VOICE; Evans et al, 2012)		x (VOICE participants)	
Warwick-Edinburgh Mental Well-being Scale (WEMWBS; http://www2.warwick.ac.uk/ fac/med/research/platform/)	x	x (including VOICE participants)	x
Health Economic (HE) Measure	x	x	x
**Staff Measures**			
Informed consent	x		
Maslach Burnout Inventory (MBI; Maslach & Jackson, 1981)	x	x	x
Views on Therapeutic Environments (VOTE; Laker et al, 2012)	x	x	x

All outcome assessors will receive training in the outcome measures from the project team and have access to supervision from a clinically qualified investigator during data collection periods. As detailed in the data management section below, all sites will also be subject to data monitoring which will include compliance with completion of outcome measures.

### Intervention dropout and study withdrawal

Withdrawal from the trial will occur if the participant decides to withdraw from the trial for any or no-stated reason, the participant loses capacity to consent during the study and is lost to follow up (unable to contact at six or nine months). However, data collected up until the point of withdrawal from the trial will be retained and included in the analysis. There is no prespecified criteria for withdrawal of the intervention from a cluster, although Step two and three of the intervention may be withdrawn upon patient request and/or if in consultation with the patient, the care team feel that the intervention is causing significant distress.

### Sample size

The recruitment target is thirty-four wards. Based on a recent survey of acute inpatient wards across the NHS, it is estimated that there will be an average of 24 beds per ward (range 18-30) giving a pool of 816 patients at baseline across 34 wards. Based on the same survey, it is estimated that there will be an average of 25 staff per ward giving a potential pool of 850 staff across 34 wards. Based on trials of psychological therapy in inpatient settings [27, 30] it has been conservatively estimated that recruitment rates for the completion of individual level outcome measures at baseline will be 80% of eligible staff and 50% of eligible patients giving a pool of 680 staff and 408 patients.

For the primary outcome of number of serious incidents on the ward, assuming 5.22 mean number of events in the control arm [31], 16 wards per arm has more than 80% power to detect a reduction in mean number of events to 3.13 (40% relative reduction), assuming a poisson distribution for the outcome. To allow for possible dropout of a ward, we will recruit 34 wards in total.

For the individual level health related primary outcome (WEBWBS) and other individual level secondary outcomes, we used the –clustersampsi- command in Stata. We assume a between group effect size of 0.4 and an intra-cluster correlation coefficient of 0.05. We allow for a baseline-endpoint correlation of 0.3, which we suggest is conservatively low given the short time span between baseline and endpoint. We specified 85% power, 5% significance level, and calculated the average cluster size (i.e. how many participants were required to be recruited in each cluster). The optimal design for these parameters requires 9 participants per cluster. This leads to a total sample size per arm of 153, and 306 for the analysis set. Accounting for attrition of 20%, by making an adjustment to this value, 383 participants are required at baseline. This assumes that attrition is consistent across treatment arms and clusters. Therefore the aim is to recruit 34 clusters with 11 participants in each cluster, giving a total sample size at baseline of 384. Adjustments will be made for additional covariates in order to preserve 85% power against a larger observed ICC value or a weaker observed baseline-endpoint correlation.

We assume a between group effect size of 0.4 for individual level health primary outcome measure (WEMWBS) and also secondary outcome measures for two reasons. First, meta-analytic reviews show that the overall effect size Cognitive Behaviour Therapy for psychosis trials is 0.4 (e.g. [13]). Second, the population norms for the WEMWBS gives a SD of 8.7, so the effect size corresponds to a between-group difference of 3.5 on the scale. According to the WEMWBS user guide (2015) investigations suggest that a change of three or more points is likely to be recognisable to an individual and different statistical methods provide estimates ranging from three points to eight points. The guide also cites a study which corroborates the importance of a change of three or more points on the WEMWBS by demonstrating correlations between score changes on WEMWBS and clinical assessment of change in the context of a counselling service; an effect only evident at three points and above. Below a three-point change, the extent of change in WEMWBS was not correlated with change assessed as clinically significant (see https://warwick.ac.uk/fac/sci/med/research/platform/wemwbs/development/papers/margerita_aucc_analysis_3.pdf).

At the 6-month follow-up where additional patients will be recruited to complete the VOICE they will also complete the primary outcome measure (WEMWBS). This is to ensure the primary outcome measure is sufficiently powered. 9 patients per cluster will be recruited, as per the aforementioned optimal design for the defined power and significance level parameters, as these participants will not receive a follow-up, we do not need to take into account drop-out rates.

### Data analysis

Analysis will follow intention-to-treat principles: outcome data will be sought and included in the analysis for all patients irrespective of receipt of the intervention during the time scale of the trial. The CONSORT statement for cluster RCT's will be followed. Treatment effects for the cluster (ward) level outcomes will be estimated using appropriate mixed models with a fixed effect for ward gender, and a random intercept for the ward.

Individual (patient/staff) level outcomes measures will be analysed using an appropriate generalised linear mixed model with random intercepts for wards. With the exception of patient ward atmosphere (VOICE), the baseline values of the outcomes can be used as a covariate in the models.

A statistical analysis plan for primary and secondary outcomes, including sub-group analyses will be presented to and agreed with the programme steering group prior to the allocation codes being revealed and commencement of any data analysis. There are no planned interim analyses.

### Health economic evaluation

This economic evaluation will be a cost-effectiveness analysis, and the primary objective is to explore the cost-effectiveness of a psychological service model designed specifically for acute mental health wards. This economic analysis will be conducted from the societal perspective incorporating costs borne by the NHS, personal and social services, criminal justice system and education authorities. This perspective is recommended by the National Institute for Health and Care Excellence (NICE) for interventions with health and non-health outcomes in the public sector and other settings [32].

Quality-Adjusted Life Years (QALYs) will be used as the health outcome measure of the cost-utility analysis. The EQ-5D-5L will be used to assess the health status and to estimate QALYs, using published EQ-5D-5L utility weights for the UK [33]. There is good evidence from previous trials of inpatients that patients can complete EQ-5D and resource use inventories during an inpatient admissions [16, 27]. The decision to use patient-reported health status as opposed to case note review has been made as the majority of patients will have been discharged from hospital at the follow ups and resource use will not necessarily be well documented or easy to locate (for example primary care services, social care services and secondary care services often use different electronic systems to record patient care). Also, previous research with mental health patients exploring differences between patient-reported health status, case note review and observations demonstrates that self-report is a reliable and objective method of collecting information on the therapeutically relevant activities and staff contacts [34, 35].

Healthcare resource use will be collected retrospectively for all included service users by means of a detailed health economic questionnaire adapted to the context of psycho-social ward-based intervention in adult acute mental health wards. The HE measures are completed by research assistants on the wards at baseline and 6 and 9 month follow-ups for both the intervention and control groups. Data collated from the health economic measure will be used to estimate the costs of health and social care service use over the past 3 months, including the current admission or any previous or future admissions and medications administered.

Medical costs that will be assessed include costs of the interventions, inpatient care and medication use. These will be estimated by multiplying resource use data by the relevant unit cost figures. Any unit costs not available for this price year will be inflated to 2019/20 prices using the Retail Price Index (RPI) [36].

Other paramount costs to consider are costs of serious incidents and costs savings resulting from reductions in staff sickness and time spent reporting serious and dealing with serious incidents at the ward level. Staff sickness is routinely recorded by wards and staff will be asked to log time spent in relation to serious incidents as part of the reporting procedure to provide estimates of typical times spent reporting and dealing with each type of incident. Unit costs for each type of service will be derived from local and national databases and statistics. Unit costs of serious incidents are not readily available and would be derived by estimating the resources that are typically used to deal with incidents arising from conflict and containment. Containment costs will be estimated based on the standard time spent by health professionals on containment measures (e.g. verbal/psychological, seclusion, physical restraints, ad-hoc medication). A micro-costing analysis of specific activities or interventions related to containment of serious incidents will be performed from the healthcare provider perspective. Information about containment measures and aggressions will be sourced from hospital databases/records (e.g. number of serious incidents or agitation episodes treated with seclusion or restraints) as it is a normal practice for a ward/hospital to maintain a register for declared serious incidents. Which, among other data, registers the number of aggression towards staff, other patients or self-harm.

Sensitivity analyses will be conducted around unit costs and numbers of incidents as well as different methods of dealing with missing data.

### Process Evaluation

The process evaluation has been designed to: (1) examine the factors that may influence the intervention delivery; and (2) explore its perceived impact and acceptability from user and provider perspectives. There are three strands to the process evaluation: assessment of intervention delivery, qualitative interviews and observational study.

### Fidelity Measures

Fidelity data will also be gathered throughout the trial period on all intervention wards. Fidelity data will be collected regarding the number of patients who were offered and received psychological assessments, formulations and therapy, at what level and over what duration. The data will be recorded by psychologists on wards that are randomised to receive the intervention, using a tool to determine whether the intervention is delivered as it should be, the quality and content. In addition, it is important to understand the type and quality of therapy being offered by the psychologist from both a patient and psychologist perspective. We will therefore be gathering data on the working alliance between patients and psychologists using the Working Alliance Inventory [37]. In addition, we will be gathering audio recordings to understand the content of what was delivered in 1-2-1 therapy sessions between the patient and psychologist. In addition to self-reporting, intervention delivering and implementation will also be assessed by independent observation. We will also be gathering audio recordings to understand the content and quality of formulation sessions between the staff and psychologist. In addition to self-reporting, the audio recordings will be listened to and assessed by an independent researcher using the Team Formulation Quality Scale (TFQS;[38]). With patient consent, psychologists on all intervention wards will record therapy sessions and 25% of all recordings will be assessed by an independent and experienced assessor on a version of the Cognitive Therapy Rating Scale for Psychosis [39] adapted for our inpatient model of therapy. Additionally, qualified nurses will detail the duration, date, focus, and number of the session when they have 1-2-1 therapy sessions with a patient. Group therapy facilitators will also document the duration, focus and number of patients in attendance to the therapy sessions. We will also gather immediate feedback from intervention wards from staff who attend TULIPS training delivered by the ward psychologist using an anonymous training feedback form.

The psychologist, on wards that have been randomised to the intervention arm, will be asked to complete a number of self-report measures to detail:

- Their daily activities each day of working on the ward.
- Each 1-2-1 therapy sessions that takes place between the psychologist and patient across the 6-month intervention period.
- Each formulation session that takes place across the 6-month intervention period (to include items from the Team Formulation Quality Rating Scale (TFQS; [38])
- Each personal supervision session that takes place across the 6-month intervention period.
- Each staff supervision session that takes place across the 6-month intervention period.
- Each training session that takes place across the 6-month intervention period.
- Each ward round that is attended by the psychologist across the 6-month intervention period.

### Qualitative Interviews

Further in-depth qualitative data will be collected throughout the trial using semi-structured interviews to enable us to capture emerging changes in implementation, such as unanticipated or complex causal pathways, facilitators or barriers to implementation. The semi-structured interviews will focus on understanding the mechanisms of action, how context affects implementation, why those delivering or receiving the intervention do not engage as planned including any unanticipated mechanisms or consequences.

### Ethnographic observations

In addition to assessing the extent to which the intervention is delivered as it is intended, it is also important to understand ‘contextual factors’ that affect delivery such as staff enthusiasm, organisational routines and resources [40]. Understanding these contextual factors is essential in informing ‘real world’ implementation of the intervention [40]. Six wards will be selected using an observation ward sampling frame. Wards will be selected based on the factors such as: gender, ward history of psychological input versus no previous psychological input, effective versus poor adoption of other ward-based psychosocial interventions, and experience of the psychologist: prior ward experience versus none. Ward managers will be asked for consent for the researcher to conduct observations on the ward. The qualitative researcher completing the observations will discuss the purpose of them with staff on the six selected wards at handovers, staff meetings and as the work is undertaken.

On each of the six wards, the researcher will undertake up to five days of observational data collection at three time points. There will be both purposeful and broader ward observations. Purposeful observations are those relating to specific events, meetings or therapy sessions on the ward. This will include attending and documenting some or all of the following: ward rounds; handover meetings; administration of medication; staff meetings; patient community meetings; and group therapy sessions. Further specific observed events may be added during the data collection. We will seek written informed consent to observe meetings and group therapy sessions from all participants present prior to the meeting/session taking place. Whilst full group consent will be aimed for, we will ensure that those who do not consent are not included in the observations.

Timings of ward observations will be specific to the ward itself, for instance when the ward rounds occur. Initial timetabling of observations will be developed in collaboration with the ward manager to ensure that such moments are captured in the data collection. A period of shadowing before the observations will also be arranged for researcher familiarisation of the ward.

Broader observations will be used to capture ward culture and environment outside of these meetings. These will occur in public spaces, such as in social areas on the wards. For these we will clearly display posters to indicate which area researchers will be observing and over what period of time so patients or staff can avoid the area if they so choose.

Data will be recorded through a field diary, and Likert scales to assess the overall ward environment when the researcher first walks on to the ward, midway through the observational day, and when leaving the ward. These will assess: how chaotic the ward is; how busy the ward is; interactions between the staff and researcher; and the level of compassion shown between staff and patients. This will be used everyday day that the ward observations occur. This will allow evaluation of the ward environment during the trial. Field diary notes will be typed as soon as possible throughout and after each data collection day. The paper notes will then be disposed of in confidential waste.

## Discussion

### Strategies to improve and monitor adherence

All psychologists (intervention deliverers) will receive two days of training in the intervention model which will be delivered by the research team. The content of the training will include an introduction to the project and intervention, training others, 1:1 therapy on inpatient settings, supervision with others, team formulation, reflective practice, data collection and reporting, and implementation challenges and will involve a combination of direct teaching and opportunities for discussion. Following the training, psychologists will receive one hour of supervision each week. This will comprise fortnightly supervision with an onsite supervisor (a senior psychologist with experience in working in inpatient setting) and fortnightly supervision with the Chief Investigator or another senior clinician from the project team. Supervision will focus on individual client work, training delivered by the psychologist, planning of groups, team dynamics, team formulation, staff support groups and any additional barriers to implementation. Adherence will be monitored throughout the intervention delivery period using a diary.

### Data management, confidentiality and sharing

Manchester Clinical Trials Unit will provide data management services. Data will be entered remotely on to a centralised web-based data capture system (RedCap) by authorised staff at participating NHS sites. The case report form captures trial data and has been specifically designed for this trial. Access to RedCap is controlled by usernames and encrypted passwords. The system provides a full electronic audit trail as well as validation features which will be used to monitor study data quality. The identity of participants will be protected by the removal of any identifiable data prior to dissemination of information, and no identifiable data will be transferred to the statistician or health economist. All participating NHS sites will be subject to data monitoring reviews to check data entry, consent and eligibility, among other items.

Trial data will be held and available for five years after the end of the trial. Data will not be archived in a repository, instead data will be released on a case-by-case basis. We shall make data available to the scientific community with as few restrictions as feasible. Data access requests will be reviewed and authorised by the Project Management Group during the trial and by the Chief Investigator after the trial has ended. Access requests will be considered against predetermined criteria and data sharing will only take place if this aligns with the consent provided by study participants. Data will be anonymised prior to being shared.

All participant data will be held on password protected computers or in locked filing cabinets at the University of Manchester or at participating NHS Trust sites. Consent forms and any other identifiable information will be kept in a separate locked filing cabinet or computer file. Access to data will be limited to members of the research team and relevant regulatory authorities. Trust and University Governance procedures will be adhered to at all times.

## Ethics, governance and safety

### Informed consent

Prior to participating in self-report measures/interviews, all potential participants will be provided with a written copy of the participant information sheet. Where possible potential participants will be given at least 24 hours to decide whether or not they would like to participate. In all instances, participants will be provided with the opportunity to read the PIS, be provided with the opportunity to ask questions about the research, and have their questions answered satisfactorily. Before beginning the self-report measures/interview, participants will be asked to sign a consent form to participate. The information sheet and consent form will state that participation is entirely voluntary and that participants are free to withdraw from the study at any point. Lack of capacity to consent will be an exclusion criterion for patients as determined by research staff in consultation with clinical teams. It is assumed that all staff will have capacity to consent to the study.

### Risks and burden to staff participants

It is important to note that participating in research may be particularly burdensome to clinicians due to busy workloads. To reduce burden associated with completing assessments, questionnaires will be limited to 30 minutes and where possible, will take place in the workplace. Interviews will be limited to one-hour duration and where possible conducted in the workplace.

### Distress and risk to patient participants

Patient questionnaire completion will be limited to 45-minute duration where possible. Furthermore, patients will be given the option to take breaks during the completion of the questionnaires, or stop anytime and complete over more than one session. It is also possible that some participants may be asked questions which they may find distressing to think about. Detailed distress and risk protocols will be put in place, detailing how to manage distress and disclosures of risk and safeguarding issues.

### Risk to researchers

In the case of patient participants, professionals involved in the patients' care will be contacted for risk information prior to all interviews and depending upon the level of risk identified the researcher will follow University and Trust guidance on safe working, including each local site lone worker policy for home visits. Researchers will also receive ongoing supervision from the chief investigator who is an experienced clinical psychologist or in the case of sites outside of the North West, other suitably qualified principal investigators.

### Governance

The sponsor of the trial is University of Manchester. The Project Management Group contains project co-applicants, programme grant project manager, members of the data management team, Trial Manager and other representatives and oversees the operation of the trial and enables communication throughout the Trial, for example, to disseminate protocol amendments. Protocol amendments will be reviewed first by the sponsor for sign off then uploaded to the online amendment system for subsequent review and approval by the relevant REC. The trial manager will then inform research sites regarding any new amendments and accompanying documents

An independent Trial Steering Committee (TSC), comprised of an independent statistician, PPI representative, a Senior Clinical Researcher and a Senior Clinician provides overall supervision of the trial, advises the CI, oversees protocol modifications, monitors the trial’s progress and if necessary closes the trial. An independent Data Monitoring and Ethics Committee (DMEC) comprised of one independent statistician and two senior Clinical Researchers, reviews the trial protocol, monitors patient safety and advises the TSC if they feel the trial should be prematurely closed.

### Safety

An adverse event (AE) and adverse reaction (AR) reporting system will be used on the trial. An SAE (serious adverse event) either: 1. results in death, 2. is life-threatening (subject at immediate risk of death), 3. requires hospitalisation or prolongation of existing hospitalisation, 4. results in persistent or significant disability or incapacity 5. is otherwise considered medically significant by the investigator. All SAEs will be assessed to see if they are related to the intervention or other trial procedures, and if they deemed related appropriate governance bodies will be informed as required. SAEs will be periodically reported to the trial’s DMEC and TSC. Additionally, we consider safety of the researchers to be extremely important and will adhere to each Trust’s lone worker policy. This generally involves the researcher carrying out a risk assessment prior to seeing the patient alone and in the case of home visits completing a form detailing information about the participant visits and their contact information and provide this to a ‘buddy’ who will ensure the safety of the researcher. The researcher must check in with the buddy before and after a visit finishes or the buddy will follow escalation procedures. Checklists provide guidance on what to do before and during the visits, for example, ensuring phones are fully charged and being prepared to leave in an emergency if there are concerns about safety.

### Auditing

The study may be subject to an audit at any time either by Manchester Clinical Trials Unit, study sponsor (University of Manchester) or Health Research Authority.

### Ancillary and post-trial care

We have a distress protocol which covers both staff and patient participants. This study specific information is part of a suite of standard operating procedures sent out to sites and research staff prior to the commencement of recruitment.

### Dissemination

The key output will be a potentially effective and cost-effective ward-based psychological intervention that increases patient access to psychological therapy in inpatient settings, is feasible to deliver in inpatient settings and is acceptable to patients.

The trial will adhere to an authorship policy in which an established writing group will determine whether or not a request for dissemination can proceed. There is a process in place to establish new methods of dissemination including new papers, reports and presentations.

The findings of the research will be widely disseminated to all relevant stakeholders, including patients, mental health staff, NHS managers, service commissioners and the general public. The study and the findings will be hosted on the University of Manchester website and the webpage will provide links to key publications and presentations. All research participants will be asked if they want to receive details of the study findings and if they consent a lay summary will be emailed or posted to them. Journal articles outlining the main findings will be written in academic journals, leading service development journals and publications aimed at frontline staff and patients.

Findings will be presented at national and international conferences targeting both clinicians and academics. Findings will also be presented at events organised by special interest groups, seminars organised by NHS and University departments and events organised by patient groups, such as Rethink and Mind. In addition, work will be undertaken with patient groups, such as Rethink and Mind to promote the study findings on their websites via links to both written and video material. Finally, work will be undertaken with the University and Trust departments for media and communication to engage with the media more generally; for example, using social media accounts, posting videos of key findings on YouTube and presenting findings as part of the Manchester Science Festival. Dissemination to patients and the wider public will be strongly guided by the patient reference group attached to the project.

All publications will seek the approval of the NIHR (funding body) and acknowledge the contribution of the NIHR, CRN, NHS and Lead NHS Trust. The full protocol and dataset from the study will be available from the Chief Investigator upon reasonable request.

## Data Availability

NA

## Abbreviations

AE: Adverse Event
AR: Adverse Reaction
BSI: Brief Symptom Inventory
CRN: Collaborative Research Network
CTU: Clinical Trials Unit
DMEC: Data Monitoring and Ethics Committee
EQ5D: EuroQol- 5 Dimension
FTE: Full Time Equivalent
IAPT: Improving Access to Psychological Therapies
ICC: Intraclass Correlation Coefficient
MBI: Maslach Burnout Inventory
MDT: Multi Displiniary Team
NHS: National Health Service
NIHR: National Institute of Health Research
NICE: National Institute of Clinical Excellence
PSP: Personal and Social Performance Scale
QALYs: Quality-Adjusted Life Years
RPI: Retail Price Index
RCT: Randomised Controlled Trial
REC: Research Ethics Committee
SAE: Serious Adverse Events
SD: Standard Deviation
SMI: Severe Mental Illness
TFQS: Team Formulation Quality Scale
TSC: Trial Steering Committee
TULIPS: Talk, Understand and Listen for In-Patient Settings
VOICE: Views on Inpatient Care
VOTE: Views on Therapeutic Environments
WEMWBS: Warwick-Edinburgh Mental Well-being Scale
